# Are hospitals collateral damage? Assessing geospatial proximity of 2000 lb bomb detonations to hospital facilities in the Gaza Strip from October 7 to November 17, 2023

**DOI:** 10.1371/journal.pgph.0003178

**Published:** 2024-10-10

**Authors:** Dennis Kunichoff, David Mills, Yara Asi, Sawsan Abdulrahim, Bram Wispelwey, Osama Tanous, A. Kayum Ahmed, Weeam Hammoudeh, Nadine Bahour, Mary T. Bassett, P. Gregg Greenough

**Affiliations:** 1 François-Xavier Bagnoud Center for Health and Human Rights, Harvard University, Boston, MA, United States of America; 2 University of California San Diego School of Medicine, La Jolla, CA, United States of America; 3 School of Global Health Management and Informatics, University of Central Florida, Orlando, FL, United States of America; 4 Faculty of Health Sciences, American University of Beirut, Beirut, Lebanon; 5 Harvard Medical School, Boston, MA, United States of America; 6 Division of Global Health Equity, Brigham and Women’s Hospital, Boston, MA, United States of America; 7 Institute of Community and Public Health, Birzeit University, Birzeit, Palestine; 8 Harvard Humanitarian Initiative, Harvard University, Cambridge, MA, United States of America; Yale University School of Medicine, UNITED STATES OF AMERICA

## Abstract

After attacks in Israel led by Hamas militants on October 7, 2023, Israel launched a major military campaign in the Gaza Strip that has featured an unprecedented scale of destruction. This has included the use of highly destructive weapons in a densely populated area. Mark-84 bombs (M-84s) are 2000 lb air-dropped explosive munitions with the capacity to damage infrastructure and kill or cause severe injury hundreds of meters away. This study examines the proximity of M-84 bomb detonations to hospital infrastructure in the Gaza Strip. We combined geospatial data on hospital locations across the Gaza Strip with maps of the locations of M-84 bomb craters between October 7 and November 17, 2023, published by CNN and New York Times. We then measured and summarized the proximity of the bomb craters to hospitals across the territory. We identified 592 M-84 bomb craters. Of the 36 hospitals across the Gaza Strip, 25% (n = 9) had at least one bomb crater within the lethal range (360 m) and 83.3% (n = 30) within the infrastructure damage and injury range (800 m) of their facilities. The shortest distance of a bomb crater from a hospital was 14.7 m. Two hospitals had as many as 23 and 21 bomb craters within 800 m of their facilities and one hospital had seven bomb craters within 360 m. Thirty-eight M-84 bombs were detonated within 800 m of hospitals in the Israeli military defined evacuation zone. Given the known blast effect of these M-84 bombs, the impact from the bomb detonations near hospitals likely killed and injured people in and around the hospital area, which could include civilians and hospital staff, and likely damaged hospital infrastructure. The results of this study suggest indiscriminate bombing in dangerous proximities to hospital infrastructure, which is afforded special protection under international humanitarian law (IHL).

## Introduction

On October 7, 2023, a group of militants led by Hamas launched an attack inside Israel killing nearly 1,200 people and taking 240 hostages. Israel responded with an aerial, maritime and ground-based military campaign that killed over 33,000 Palestinians and injured 75,000 more in the Gaza Strip between October 2023 and early April 2024 [1]. Approximately two-thirds of those killed have been women and children [2]. Israel has since been called on by the International Court of Justice to halt all acts prohibited under the Genocide Convention, which includes killing and causing serious bodily and mental harm to Palestinians in the Gaza Strip [3].

After Hamas won the 2006 Palestinian elections and took power in the occupied Gaza Strip, Israel imposed a siege on the Strip that tightly controls any movements of people and material in and out of the territory. Between 2007 and 2023, Israel had launched four other major wars on the Gaza Strip that killed thousands of civilians and caused enormous destruction to civilian infrastructures, including health facilities, generating a “biosphere of war” [4].

Yet the scale and intensity of the Israeli military’s current bombardment campaign and the subsequent damage are considered unprecedented [5]. This current Israeli military campaign has been the most deadly and destructive of all its previous wars on Gaza combined [6], with the most killing per capita in 100 days that the world has seen since the Rwandan genocide [7]. On December 14, 2023, several news agencies reported on an assessment conducted by the United States (US) Office of the Director of National Intelligence estimating that by mid-December, the Israeli military had dropped over 29,000 bombs on the Gaza Strip since October 7, half of them being low-precision and highly destructive munitions [8]. At that point, Israel’s military campaign had already destroyed 70% of homes across Gaza and most of the electrical, water, communication, and healthcare infrastructure across the entire territory [9].

Among the munitions being used are 2,000 lb (907 kg) air-dropped Mark-84 (M-84) bombs, colloquially known as “bunker buster bombs” [9], manufactured by General Dynamics Ordnance and Tactical Systems for the US Department of Defense [10]. They are the largest among the M-80 Series Bomb Bodies, originally developed in the 1960s during the Vietnam War [11], and are typically used in bombing operations where maximum blast and explosive effects are desired. Subsequent technology can transform them into guided weapons. The shattering of the bomb’s steel casting upon detonation shoots 1,000 lbs (453 kg) of white-hot steel fragments in all directions at 1.8 km (6,000 feet) per second; these flying fragments are lethal as far as 360 m (1,200 feet) away from the point of detonation and can travel further than a mile away [12]. The explosion creates a shock wave exerting thousands of pounds of pressure per square inch (psi) that can displace 10,000 lbs of dirt, forming a crater in the ground up to 15 m (50 feet) wide and 11 m (36 feet) deep. For comparison, a shock wave of 12 psi will knock a person down, and 15 psi is considered the injury threshold. This fragmentation pattern and shock wave can be expected to cause severe injury, including rupturing lungs, bursting sinuses, and tearing off limbs [13], and may damage infrastructure as far as 800 m away from the point of detonation.

Given their highly destructive impact, the use of M-84 bombs during wars that involve civilian populations and infrastructure raises critical questions about violations of international humanitarian law (IHL). IHL governs conduct during armed conflict and establishes four fundamental principles aimed at limiting the destruction and suffering caused by such conflicts. First, the principle of humanity, which is informed by the dictates of public conscience; second, the principle of distinction between civilians and combatants, and between civilian objects and military targets; third, the principle of proportionality, which seeks to limit damage caused by military operations; and fourth, the principle of military necessity, which seeks to balance a legitimate military purpose with humanitarian exigencies [14]. Given the special protections afforded to hospitals under IHL and their high value function within civilian society in wartime and after, it is crucial to understand the mechanisms by which they have been damaged in the assault on Gaza in order to devise more robust legal, ethical, and material protections going forward.

While damage to hospitals has been extensively documented and reported in the current Israeli military campaign in the Gaza Strip, clarity on the strikes, locations of the bombings relative to civilian infrastructure, and the use of highly destructive weaponry remains limited. Satellite imagery analysis can be used as a methodologic tool to assess the current damage and to monitor the impact of the ground-based military campaign [15]. The high volume of news media satellite imagery investigations examining the impacts of the war offers valuable data that can be used to examine patterns of destruction and provide insight into questions around compliance with IHL.

While the Israeli military possesses and uses a diverse and extensive artillery, in this paper we have chosen to focus on the 2000 lb bombs because of their signature craters as well as the magnitude of damage those bombs inflict within a large radius on structures in densely populated human environments. Thus, damage to structures and injuring or killing of humans within the lethal range is not truly “collateral” but is, in reality, an anticipated effect of those bombs when chosen over smaller munitions. By using publicly available maps locating the detonations of 2000 lb bombs published in news media, the objectives of the study include the following: (1) to assess the proximity of 2000 lb M-84 bomb craters to hospitals across the entire Gaza Strip; (2) to interrogate the utility of bomb crater data on questions surrounding the special protections afforded to hospitals under IHL.

## Methods

We utilized publicly available geospatial data to identify hospital sites and combined that with geospatial data extracted from CNN and New York Times (NYT) satellite imagery investigations to describe the number and proximity of 2,000 lb M-84 bomb craters to hospitals in the Gaza Strip.

CNN’s investigation [16] in collaboration with Synthetaic (a US-based artificial intelligence [AI] company), published on December 22, 2023, detected more than 500 craters at least 12 m (39 ft) wide in the two northern governorates of the Gaza Strip (Gaza and North Gaza, above the Wadi Gaza demarcation line). Four satellite images taken from October 15 to November 6 were used for their analysis. The authors utilized an AI algorithm developed by Synthetaic, called RAIC (Rapid Automatic Image Categorization), which uses unsupervised machine learning methods to detect the bomb craters across the satellite imagery. Three members of the authorship team, including one from Synthetaic and two open-source intelligence journalists from CNN, then manually reviewed the results. The authors do not state the exact final number of bomb craters they mapped. In their online article, CNN provides a map displaying the bomb craters they identified.

NYT published an 8:34 minute video investigation [17] on December 21, 2023, reporting on destruction from munitions in the Israeli-designated evacuation zone of the Gaza Strip. This investigation, conducted in collaboration with Planet Labs (a global provider of satellite imagery), also uses satellite imagery data to identify and quantify the number of 12 m (39 ft) wide craters in the governorates below the Wadi Gaza demarcation line (Deir Al-Balah, Khan Younis, and Rafah). The two investigations’ geographic regions show no overlap. The NYT journalists first used the platform Picterra to train an algorithm with images of bomb craters from Gaza that were roughly 40 ft, in this case utilizing supervised machine learning methods. They ran three iterations of the algorithm on images of southern Gaza from November 16 and 17 and then manually assessed these results to remove false positives. They state a final count of 208 M-84 bomb craters selected in the process, a likely undercount given that the satellite images did not cover the entire surface area of southern Gaza, among other limitations common to this type of satellite imagery analyses. A co-author of the investigation describes their methodological process in detail on the social media platform X (formerly Twitter) [18]. On the video publication page, at minute 1:31 of the video, a map of the bomb craters identified by satellite imagery across their geographic area of focus is presented.

We imported the two screenshot images of the maps produced by the media investigations as raster data files into ArcGIS Pro 3.2 (ESRI, Redlands, CA). To assign geographic coordinates to our screenshots, we used ArcGIS Pro’s georeferencing tools to connect the raster data of our screenshots to an open-source map with a WGS 1984 UTM Zone 36N geographic coordinate system. Geographically linking the raster to the map was done by matching features from both the image and the map, such as state borders or major road networks. Once the image was positioned so that its features overlapped with the corresponding features on the map, coordinates of the image and its features were identified and made into a geospatial dataset for analysis.

Using data from the UN Office for the Coordination of Humanitarian Affairs (UN-OCHA) and Open Street Map (OSM), we generated a geospatial dataset of pre-October 7 hospital areas across the Gaza Strip. The UN-OCHA dataset titled “Gaza–Hospitals and Health Centers” provided a list of all 36 hospitals in the Gaza Strip, with their names in English and Arabic, the number of beds, and their geographic coordinates, found under the Excel sheet tab “Hospitals” [19]. These data represented spatial points but contained no information on the structural size and shape of the facility area. We generated these features using geospatial data creation tools in ArcGIS Pro that allow users to manually define polygon shapes and sizes. Specifically, we created the hospital area polygons by capturing the entire building footprint of buildings in the OSM base-map layer that the spatial point data intersected with. We also used UN-OCHA data to describe the number of beds in each hospital as a function of hospital size and treatment capacity.

We combined the two sets of georeferenced data to perform spatial summaries of the bomb craters and hospitals. We measured the closest distance of a bomb crater and the number of bomb craters within 360 m, the ‘lethal’ range, and 800 m, the ‘damage and injury’ range, to each hospital. We used the centroid of the crater for all of our measurements. We describe these measures with summary statistics in tables and a figure as well as in descriptive maps. Statistical summaries were calculated using R Statistical Software (v4.2.2; R Core Team 2022).

The study was deemed not involving human subjects and granted an exemption by the Harvard University Longwood Campus Institutional Review Board’s decision tools.

## Results

**Fig 1:**
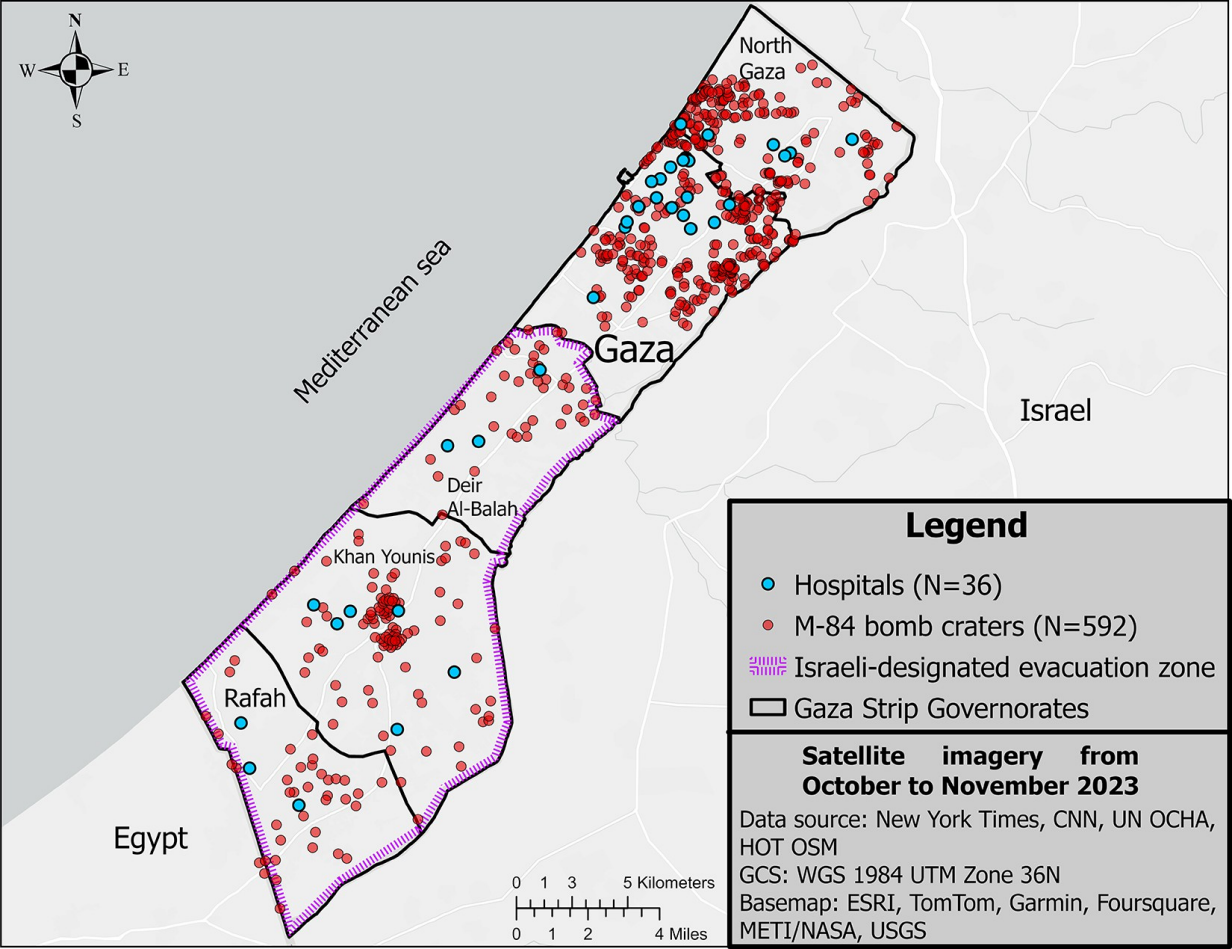
Proximity of bomb craters to hospitals and within the Israeli military designated evacuation zone in the Gaza Strip.

Georeferencing the two media investigations produced a map file of 592 craters formed by M-84 bombs from October 7 to November 17, 2023 (Fig 1). CNN’s data, which focused on the northern two Gaza governorates, accounted for 70.1% (n = 415) of the craters identified. NYT’s data, which focused on the three governorates south of the Wadi Gaza demarcation line and the evacuation zone designated by the Israeli military, accounted for 29.9% (n = 177) of the craters identified.

**Fig 2:**
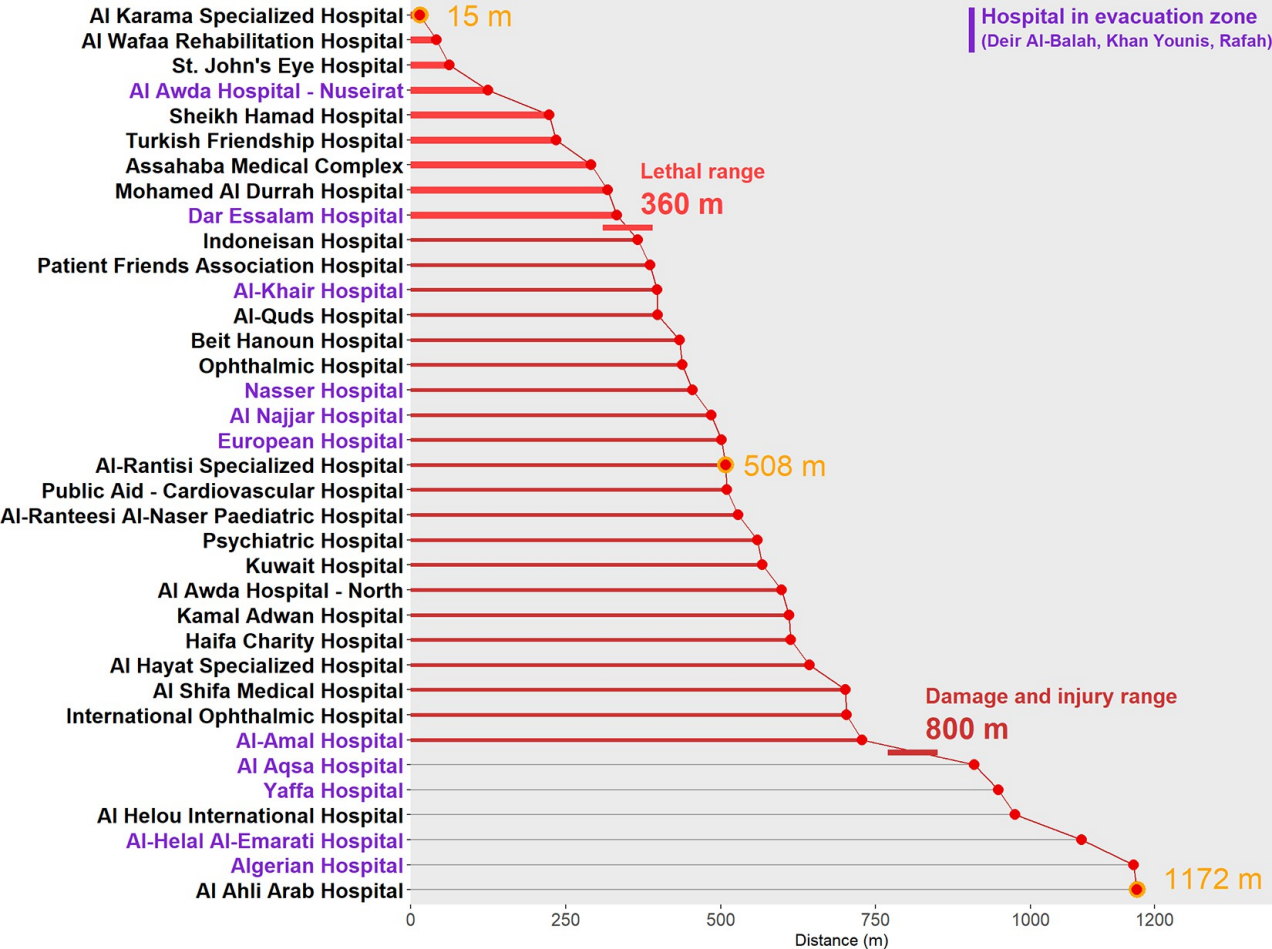
Distances between hospitals and most proximal bomb crater for all hospitals in the Gaza Strip.

A total of 36 hospitals and their area boundaries were identified and defined from the OCHA and OSM geospatial datasets (S2 Data (hospitals), S1 Data Dictionary): 16.7% (n = 6) in the North Gaza governorate, 50% (n = 18) in the Gaza City governorate, and 33.3% (n = 12) in the evacuation zone (8.3% (n = 3) in Deir Al-Balah, 16.7% (n = 6) in Khan Younis, and 8.3% (n = 3) in Rafah). All 36 hospitals in the Gaza Strip had at least one bomb crater within 1,200 m of the facility (Fig 2). The shortest distance of a bomb crater to a hospital facility was 14.7 m for all hospitals across the Gaza Strip and was 124.4 m for hospitals in the evacuation zone. The median distance of a bomb crater to any hospital facility was 508.4 m and 75% (n = 27) of the hospitals had a crater within 657.6 m.

**Fig 3:**
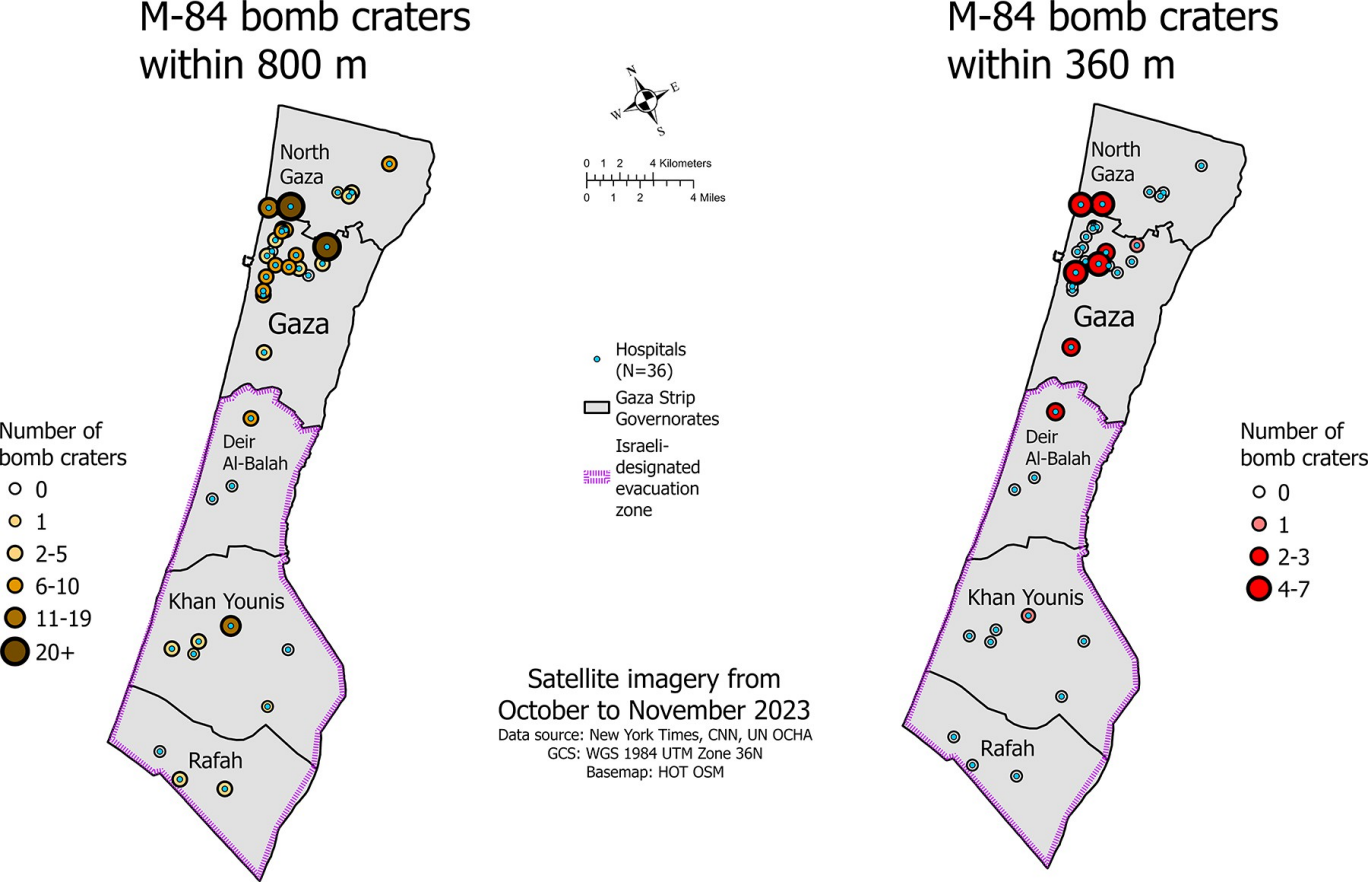
Hospital locations and the number of bomb craters within 800 m and 360 m of hospitals.

Table 1 provides details on the number of beds, the closest crater, and the number of craters within dangerous proximities of each hospital. Table 2 summarizes the counts and proportions of bomb craters in dangerous proximities to all hospitals across the Gaza Strip. Fig 3 presents a mapped summary of the data. Al Karama Hospital in North Gaza, Mohamed Al Durrah Hospital in Gaza, and Dar Essalam Hospital in Khan Younis had the most bomb craters within 800 m, the ‘damage and injury’ range, of their facilities: 23, 21, and 18 bomb craters, respectively. Within the 360 m ‘lethal’ range, 25% (n = 9) hospitals across the Gaza Strip, including Dar Essalaam Hospital in the evacuation zone, had one or more bomb craters, and 11.1% (n = 4) hospitals had three or more bomb craters. The largest hospital across the Gaza Strip, Al Shifa Medical Hospital, had two bomb craters within 800 m of its facilities and the second largest hospital, Nasser Hospital, had a bomb crater 454 m from its facilities. Across the entire Gaza Strip, 83% (n = 30) of hospitals had at least one bomb crater within 800 m. In the northern governates, 91.7% (n = 22) of hospitals had at least one bomb crater within 800 m as did 66.7% (n = 8) of hospitals in the evacuation zone. Altogether, bomb craters within 800 m and within 360 m of hospitals accounted for 33.3% (n = 197) and 4.7% (n = 28), respectively, of the 592 georeferenced craters (S3 Data (bomb craters)).

**Table 1:** Number of beds, closest bomb crater, number of bomb craters within 360 m, and number of bomb craters within 800 m for each hospital by governorate in the Gaza Strip.

Governorate	Name	Number of beds	Closest crater (m)	Number of craters within 360 m	Number of craters within 800 m
North Gaza	Al Awda Hospital—North	75	597.9	0	4
	Al Karama Specialized Hospital *** ‡‡	40	14.7	4	23
	Beit Hanoun Hospital *	86	433.5	0	6
	Indonesian Hospital	134	365.9	0	3
	Kamal Adwan Hospital	89	610.0	0	1
	Sheikh Hamad Hospital for Rehabilitation and Prosthetics ** ‡‡‡	40	222.8	7	18
Gaza	Al Ahli Arab Hospital	80	1172.0	0	0
	Al Hayat Specialized Hospital	40	643.0	0	2
	Al Helou International Hospital	60	974.8	0	0
	Al-Quds Hospital *	102	398.3	0	6
	Al-Ranteesi Al-Naser Paediatric Hospital *	215	527.6	0	6
	Al-Rantisi Specialized Hospital *	99	508.4	0	7
	Al Shifa Medical Hospital	744	701.6	0	2
	Assahaba Medical Complex * ‡	24	290.9	2	8
	Al Wafaa Rehabilitation Hospital * ‡‡	70	41.1	4	8
	Haifa Charity Hospital	26	613.3	0	2
	International Ophthalmic Hospital *	43	702.7	0	6
	Mohamed Al Durrah Hospital *** ‡	80	317.4	1	21
	Ophthalmic Hospital *	40	437.5	0	7
	Patient Friends Association Hospital *	54	385.9	0	7
	Psychiatric Hospital *	46	559.5	0	7
	Public Aid—Cardiovascular Hospital *	30	509.9	0	5
	St. John’s Eye Hospital * ‡‡‡	10	61.7	5	7
	Turkish Friendship Hospital ‡	272	234.2	2	3
Deir Al-Balah	Al Aqsa Hospital	304	909.2	0	0
	Al Awda Hospital–Nuseirat * ‡	60	124.4	2	8
	Yaffa Hospital	30	947.8	0	0
Khan Younis	Al-Amal Hospital	100	728.1	0	2
	Algerian Hospital	5	1167	0	0
	Al-Khair Hospital	15	397.0	0	3
	Dar Essalam Hospital ** ‡	35	332.2	1	18
	European Hospital	290	500.7	0	1
	Nasser Hospital	475	454.0	0	1
Rafah	Al-Helal Al-Emarati Hospital	75	1083.0	0	0
	Al Najjar Hospital	102	484.8	0	3
	Kuwait Hospital	16	567.4	0	2

* 5 or more craters within 800m

** 10 or more craters within 800m

*** 20 or more craters within 800m

‡ 1 or more craters within 360m

‡‡ 3 or more craters within 360m

‡‡‡ 5 or more craters within 360m

**Table 2:** Counts of hospitals with bomb craters in dangerous proximities by region in the Gaza Strip.

		All hospitals in the Gaza Strip (n = 31)	Hospitals in northern Gaza Strip (North Gaza and Gaza) (n = 21)	Hospitals in the Israeli military designated evacuation zone (Deir Al-Balah, Khan Younis, Rafah) (n = 10)
Bomb craters within 360 m	Any craters	9/36 (25%)	7/24 (29.2%)	2/12 (16.7%)
	Number of craters			
	1	2	1	1
	2–5	6	5	1
	6–10	1	1	0
	11–19	0	0	0
	20–23	0	0	0
Bomb craters within 800 m	Any craters	30/36 (83.3%)	22/24 (91.7%)	8/12 (66.7%)
	Number of craters			
	1	3	1	2
	2–5	11	7	4
	6–10	12	11	1
	11–19	2	1	1
	20–23	2	2	0

## Discussion

Our findings demonstrate that 84% of all hospitals were within 800 m, the ‘damage and injury’ range, of at least one M-84 bomb detonation, and 25% of all hospitals were within 360 m, the ‘lethal’ range. Hospitals within these ranges are expected to have sustained infrastructural damage and human casualties from these highly destructive bombs, findings in accordance with reports from the WHO, UN OCHA, and Palestinian health institutions and professionals [20, 21].

### Damage to civilian infrastructure in IHL

This study adds to the body of evidence delineating the overwhelming damage to civilian infrastructure in the Gaza Strip during the Israeli military campaign, including hospitals, which are afforded special protected status under IHL [22–24]. Hospitals only lose their protection from attack if they are being used to commit “acts harmful to the enemy.” However, even in these cases, the attacking force must issue a warning, set a reasonable time within which these acts must end, and may only lawfully attack after such a warning is disregarded [25, 26]. Furthermore, attacks on hospitals are unlawful if indiscriminate or disproportionate, an inevitable feature of large explosives used in a densely populated area. IHL protections for hospitals are critically important because even the threat of an attack or minor damage can have profound implications for the provision of health care. While we did not identify bomb craters directly on hospital facilities, the proximity of bombs detonated within dangerous ranges to hospital facilities, raise concern for violations of IHL.

### Asymmetrical warfare and the use of M-84 bombs

Modern wars have increasingly taken the shape of asymmetrical warfare between states with formal armies fighting non-state actors without formal armies. When this type of asymmetrical warfare targets highly dense urban areas, the damage to civilian populations and infrastructures can be overwhelming. In this type of asymmetrical counter insurgency warfare [27, 28], Palestinian occupied territories become sites for various types of warfare, such as “almost war” in the West Bank [29] and siege warfare in the Gaza Strip [30], with periods of intense asymmetrical wars. The previous four Israeli wars on Gaza in 2008, 2012, 2014 and 2021 have all been characterized by the Israeli military use of the “Dahiya Doctrine”, which justifies disproportionate force and causing great destruction to an area including civilian infrastructures [31]. The Israeli military’s use of M-84 bombs in these previous wars has provoked criticism for the indiscriminate nature of the bombings, for the killing of civilians, and for the destruction of infrastructure [32]. In 2022, 83 countries, including the United States but not Israel, committed to “refraining as appropriate, from the use of explosive weapons in populated areas, when their use may be expected to cause harm to civilians or civilian objects.” [33] On October 31, 2023, the Israeli military dropped at least two M-84 bombs on the Jabaliya refugee camp [34], one of the most densely populated residential areas in the Gaza Strip.

Our analysis geolocated 592 craters formed by M-84 bomb detonations across the Gaza Strip, one of the most densely populated places in the world, in just the first six weeks of the military campaign. A third of the M-84 bomb craters (197/592) we identified were within ‘damage and injury’ range (800 m) of most hospitals across the Gaza Strip. These bombs were detonating across a geographic area which, when combined, represents 14% of the entire area of the Gaza Strip. Approximately 5% (28/592) of the bomb craters were within ‘lethal’ range (360 m), a combined area that represents 3.5% of the Gaza Strip. During the study time period, hundreds of thousands of internally displaced civilians sheltered and sought treatment in these hospitals and surrounding areas [35]. On October 18, 2023, the Gaza Health Ministry reported that Al Karama Hospital in North Gaza was no longer operational due to bombardment [36]. Our analysis found 23 craters within the ‘damage and injury’ range of this hospital, four of those being within the ‘lethal’ range, and one as close as 15 m away. Approximately one week later, Israeli military warplanes struck on and near the Turkish Friendship Hospital [37], the only specialized cancer treatment center in the Gaza Strip, and we found two M-84 bomb craters within ‘lethal’ range and three more within ‘damage and injury’ range of this hospital. It was also reported that bombs were being dropped as close as 50 m away from Al-Quds Hospital in the Gaza governorate, which was operating at four times its capacity and sheltering 12,000 displaced civilians [38]. Our analysis found six M-84 bomb craters within ‘damage and injury’ range of Al-Quds, with one as close as 398 m. Al-Shifa Hospital, the largest hospital in the Gaza Strip, where tens of thousands of civilians also sought shelter and received trauma care [39], sustained two M-84 bomb detonations within the ‘damage and injury’ range.

### Impact on hospitals in the designated evacuation zone

Although the Israeli military ordered civilians in the North Gaza and Gaza governorates to evacuate to “safe zones” south of the Wadi Gaza demarcation line, we found 38 M-84 bomb craters within lethal and damage and injury ranges of hospitals across the evacuation area. Al Awda Hospital-Nuseirat in Deir Al-Balah sustained eight M-84 bomb detonations within 800 m, with one as close as 124 m away. On November 1, 2023, patients and internally displaced people evacuated, under fire, from the Turkish Friendship Hospital in the Gaza governorate to Dar Essalam Hospital in Khan Younis, resulting in the death of four cancer patients following the evacuation [40]. Our analysis found 18 bomb craters within ‘damage and injury’ range and one within ‘lethal’ range of Dar Essalam. Nasser and European Hospitals in Khan Younis were also planned evacuation locations for patients at other hospitals. Our results show that an M-84 bomb detonated 464 m away from Nasser Hospital, the second largest hospital in the Gaza Strip, which had already been sheltering thousands of displaced people and operating at a minimum of three times its capacity [41]. We also found an M-84 bomb detonated 500 m away from the European Hospital. In Rafah, the governorate furthest south of the Wadi Gaza, our analysis found five bomb craters within ‘damage and injury’ range of hospitals located there. These findings further add to the reality that “no one and nowhere is safe” [42] in the Gaza Strip.

### Damage to hospitals and disregard of IHL

The proximity of 2,000 lb M-84 bomb craters to hospital areas demonstrates that hospitals, and by extension, their medical staff, patients, and the internally displaced people sheltering in and around these facilities, have sustained damage as a result of Israeli military strikes. The Israeli military, and its primary weapons suppliers–the US, UK, and Germany—have ready access to the coordinates of hospitals and are privy to the munition’s characteristics of M-84’s, specifically their blast radius. The targeting of hospitals, whether through direct impact or indirectly due to damage to their vicinity or access, is prohibited in IHL. Considering the limited evidence of certain hospitals actually harboring enemy militants, human rights experts contend that the Israeli military has failed to adhere to the protocols mandated by IHL for appropriate action in those situations [43]. Furthermore, our analysis illuminates the extent to which the military’s bombing campaign has endangered hospitals that have not been alleged to have imminent military threads. Importantly, damage to sites and infrastructure surrounding hospitals, such as roads, electricity, or water facilities, would impact hospital access in these settings. These findings add to the developing evidence that hospitals are not being afforded special protection in the Gaza Strip.

### Study limitations

Methodologically, as this paper relies on satellite data at distinct time points and strictly the appearance of a bomb crater in the ground, we cannot causally correlate casualty data, hospital structural damage, or hospital functionality to these munition events. We mapped close to 600 bomb craters by georeferencing publicly available maps, but approximately 100 total craters from CNN’s “over 500” and NYT’s 208 M-84 bomb craters identified were missing. These additional bomb craters were likely clustered and non-discernable in the satellite imagery maps published by CNN and NYT. Importantly, the media investigations may have missed additional bomb craters due to affected quality of satellite imagery observations as a result of cloud cover, rubble covering the targets, or low resolution. The maps published by CNN and NYT also enlarged the points representing the bomb craters to sizes slightly larger than their actual size. Therefore, the locations of bomb craters we georeferenced in this analysis may be within a few meters from the actual point of detonation, but likely by a negligible difference when considering ‘lethal’ and ‘injury and damage’ ranges to hospitals.

Due to the rate of destruction in the Gaza Strip since the start of the war, the data presented no longer reflects the current situation on the ground, which is much worse than what our findings suggest. Further, this study engages with one specific form of attack on healthcare facilities using one specific weapon. The damage inflicted on the healthcare in Gaza Strip includes, and is not limited to, depleting hospitals of equipment, medications, fuel and electricity due to the blockade, snipers placed around and within hospitals, kidnapping, torture and killing of medical staff, raiding hospitals and killing patients and staff and turning hospitals into battlefields and their yards into mass graves. The cumulative attacks on healthcare are beyond the scope of this article but can be found in emerging evidence by journalists and human rights groups [44]. In order to form a clear and complete image of the scale of attacks on healthcare in Gaza, scholars must consider all of these different pieces together. The attacks on healthcare in Gaza has been seen by some scholars as an integral part of the charge of genocidal intent [45].

## Conclusion

Analysis of geospatial bomb crater damage and hospital locations indicate highly destructive 2000 lb M-84 bombs air-dropped by the Israeli military resulting in bomb craters within ‘lethal’ and ‘damage and injury’ ranges of the majority of hospitals throughout the Gaza Strip. Given the proximity of these bomb craters to hospital areas, this study reveals concern for indiscriminate bombing in close proximity to hospital infrastructure which is afforded special protection under IHL. Given the destruction to hospital infrastructure due to these bombs, these findings support evidence that hospitals are not being afforded special protection, as mandated by IHL.

## Supporting information

S1 Data Variable definitions and descriptions of S2 and S3 Data. (DOCX)

S2 Data Information on each hospital used in the analysis. (XLSX)

S3 Data Information on each bomb crater used in the analysis. (XLSX)
