# Supplementary material for: Are hospitals collateral damage? Assessing geospatial proximity of 2000 lb bomb detonations to hospital facilities in the Gaza Strip from October 7 to November 17, 2023
Source: PLOS Glob Public Health. 2024 Oct 10;4(10):e0003178. doi: 10.1371/journal.pgph.0003178 (PMC11466298; doi:10.1371/journal.pgph.0003178)
Supplement: S1 Data — (DOCX) [file pgph.0003178.s001.docx]

**File name: S2_Data.xlsx**

**Title: Gaza Strip Hospitals with M-84 Bomb Crater Statistics**

**Description: This dataset contains information for each of the 36 hospitals in the Gaza Strip that were used for this analysis. This includes hospital information extracted from the OCHA data source (referenced in the Methods), such as names and number of beds, and also statistics describing proximity of bomb craters.**

| **Variable name** | **Definition** |
| --- | --- |
| name_eng | Hospital name in English (source: OCHA) |
| name_arabic | Hospital name in Arabic (source: OCHA) |
| hosp_longitude | Hospital longitude coordinates in decimal degrees (source: OCHA) |
| hosp_latitude | Hospital latitude coordinates in decimal degrees (source: OCHA) |
| gov | Gaza Strip governorate that the hospital is in |
| num_beds | Number of beds in the hospital (source: OCHA) |
| closest_bc_dist_m | Closest M-84 bomb crater found to the hospital |
| closest_bc_id | ID number of the closest M-84 bomb crater found to the hospital (matches IDs of the bomb crater dataset) |
| bc360m | Number of M-84 bomb craters within 360 m of the hospital |
| bc800m | Number of M-84 bomb craters within 800 m of the hospital |

**Dataset description: 36 observations; 10 variables**

**File name: S3_Data.xlsx**

**Title: All Georeferenced M-84 Bomb Craters in the Gaza Strip**

**Description: This dataset contains each M-84 bomb crater that was geolocated through the process of georeferencing the CNN and NYT bomb crater maps.**

| **Variable name** | **Definition** |
| --- | --- |
| source | The news agency source of the screenshot used to locate the M-84 bomb crater |
| id | Unique ID for each M-84 bomb crater |
| gov | Gaza Strip governorate |
| longitude | M-84 bomb crater longitude coordinates in decimal degrees |
| latitude | M-84 bomb crater latitude coordinates in decimal degrees |

**Dataset description: 592 observations; 4 variables**
